# Cerebro-Spinal Meningitis

**Published:** 1867-09

**Authors:** F. R. Payne

**Affiliations:** Marshal, Ill.


### CHICAGO MEDICAL EXAMINER.

N. S. DAVIS, M.D., EDITOR.

VOL. VIII. SEPTEMBER, 1867. NO. 9.

### Original Contributions.

ARTICLE XXXVII.

### CEREBRO-SPINAL MENINGITIS.

BY F. R. PAYNE, M.D., Marshall, Ill.

Read to the Clark County Medical Society, July 1st, 1867.

This is a disease with which you are all painfully and practically familiar. It has in the last few years prevailed in many parts of our own county. Our observations clearly demonstrate that we have at least two varieties of this fearful malady, *simple and malignant.*

For the purpose of bringing this disease fully before you, we propose to report two cases of the malignant form, which occurred in one family during the last month, one of which was examined after death.

CASE I. *May 29th, 1867, 3 o'clock P.M.* Dr. R. F. Wil-Williams and I were called to see Miss C., aged 8 years, and and found her laboring under the following symptoms: She was well this morning; ate her breakfast; about 10 or 11 o'clock A.M. she complained of being chilly, and had a severe pain in the back of her head, and in her neck, extending down the spine. The pain in head carefully noted, and did not extend above tentorium; the pulse 160; very small, and the surface of the body presented a cyanotic hue; a prickling and tingling sensation in the arms; body very sensitive and hot; eyes suffused; mind excited; speaks loud, quick, and distinct; extremities tremulous; gasping breathing; very restless; copious perspiration; tongue whitish, or creamy coat, and moist; tremulous sensation in epigastrium, and, in fact, all of the premonitory symptoms of convulsions; evidently irritation or congestion of cerrebellum and spine. These were the symptoms three or four hours after the first chilly sensation.

*7 o'clock P.M.* Nausea and occasionally efforts to vomit; violent delirium; pupils largely and permanently dilated; extremely restless; right side more effected than left; total loss of consciousness; surface cold; no pulse at the wrist; no action on bowels; rose colored spots, from the size of a pin's head to half an inch and over in diameter; not confluent, and no elevation of the skin; the spots seem to be the broken corpuscles of blood retained in the capillaries; the whole surface on back and chest blue; the spots are constant in all malignant cases; the cyanotic hue of back chest, lips, and eye-lids, we have found present in all violent cases, and after death this condition is increased. All of the symptoms clearly indicate a point with positive certainty to the cerebellum and spinal cord as the seat of disease; she breathes hard, rather slow, and laborious; twitching of the muscles of extremities, and rigidity of the extensors of the back and flexors of fingers and forearms; trismus, or partial closure of the jaw, and difficult deglutition. This was a young, healthy girl, very large for her age; full of blood and life twelve hours ago.

*11 o'clock P.M.* Symptoms about the same; every evidence of approaching dissolution; complete coma; pupils very large, and not sensitive to light; convulsive movements of right arm and leg; cold and clammy perspiration; her medicine had operated finely on the bowels; breathing rather slow and laborious. It is almost impossible for her to swallow water.

She lived until about 12 o'clock on the 30th, and then died without a struggle. She lived twenty-four hours after the first inception of the disease. The remedial agents used in this case did not seem to exert the least beneficial or controlling influence.

CASE II. *June 3d, 1867.* Another child of the same family, was seized with the same train of symptoms, about 10 o'clock to-day—a boy six years old. When first visited, one or two hours after he complained of being chilly, the symptoms were the same as the first case—if any difference they were more aggravated.

*3 o'clock P.M.* He is very restless; pupils dilate largely, and then contract; pulse very rapid; skin hot; eyes suffused; tonic spasm of muscles of the superior extremities.

*5 o'clock P.M.* Surface cold; no pulse at the wrist; pupils largely dilated; cold, clammy perspiration; complete loss of consciousness; in short, nearly the same symptoms as in the first case; the only exception is that about 8 o'clock P.M., this little boy began to halloo and scream terrifically, and continued this until midnight, and then expired.

We have had the misfortune to witness many cases of this awful disease, and feel satisfied that there are more than one variety. At least 96 per cent. of the *malignant* cases die. We are also convinced that many cases are called "spotted fever," in which the cerebro-spinal symptoms are purely sympathetic, caused from reflex action.

For the purpose of more fully bringing this important subject before the Society, we will present some important facts contained in the able, pointed, and valuable treatises on this disease by Prof. J. S. JEWELL. His paper is prepared with great care, and evinces profound research. It is published in the Chicago MEDICAL EXAMINER for October and November, 1866.

Prof. JEWELL has consulted nearly all the writers on this disease in Europe and this country, and the weight of testimony is that it is dependent upon a primary specific epidemic cause, the true nature of which is not known, save in its effects. It may not be external, but internal, or autopathic. Many external causes, singly and combined, have been assigned to this malady. Social misery, foul air, imperfect nutrition, exposure to cold and damp atmosphere, abuse of alcohol, and a host of others, have been pointed out by able medical men, singly and combined, as not only predisposing, but positive and essential causes. It is known that these external causes do not always produce the disease, it is therefore reasonable to say that it must be assigned to that indefinite and imperfectly understood class of *epidemic causes.*

Our experience accords with Prof. JEWELL. No age is exempt, but the young are the principal victims. Generally it attacks the young, vigorous, and athletic, and it is a pretty well established fact that males are more liable than females. There is no evidence that a hereditary influence plays any part in the production of this disease as an essential cause. It is a matter of much regret that there has not been more frequent examinations of the blood. It has been positively demonstrated that this disease quickly destroys the nervous, vascular, and secretory functions. The capillary congestion is rapidly followed by the developement of serous or purulent infliltration wherever local hyperæmia or inflammation exists. This being true, we feel confident that future investigations will demonstrate a breaking up of the affinities between the blood corpuscles and organized structures of the body. It is very evident that the cause, whatever it may be, of this terrible malady, has a powerful septic or degenerative tendency.

It is supposed by many that the cause of this disease is identical with that of malignant erysipelas in its essential nature. Others think it is a malignant form of scarlatina and typhus. The causes of diptheria and hog cholera are by some supposed to be identical with the cause of this disease. The attack is sudden, and marked; the progress to a fatal termination so rapid; the terrible shock to the nervous system, as shown by the symptoms, all clearly indicate that some powerful poison has been introduced into the system. That the constituents of the blood are changed from their normal condition, is also true, and no external visible primary cause can be assigned for this rapid transition from life to death. We are forced to the belief that some potent, uniform, and yet unknown agent produces the disease.

Dr. JEWELL is of the opinion that it is a disease *sui generis*, that is essentially different from, and independent of other recognized forms of disease.

Our observations favor the opinion that the agent that produces "spotted fever" is similar, if not the same, that primarally engendered "black tongue," a terrible epidemic that prevailed in this county in 1845–46.

It is almost universally the opinion of men who have had experience in this affection that it is not contagious. It does not conform to a single law of contagion, but does fully conform to the laws of epidemic influence.

The poison, whatever it may be, frequently acts directly on the nerves, but it is not yet known whether it is the changes in the blood or the direct effects of the poison that gives rise to the symptoms and appearances presented in this disease.

The *post mortem* appearances in the second case above reported will be of interest to many. Drs. Gard, Price, Williams and Lodge were present. After removing the cranium we found the diploë of the occipital bone, deeply colored with blood. This was not the case in the other bones through which the saw passed. The dura mater in the back part and base of the brain was in a high state of congestion; in fact, all the meninges in that region of the head and upper part of the spinal cord were in the same condition. The capillaries seemed enlarged and intensely engorged with blood. After being thoroughly soaked in cold water they still presented that red and engorged appearance. The tentorium was also very much engorged: beneath this membrane in cerebellum and around the pous Variola and medulla oblongata we found fully *three ounces* of a reddish serous exudation. There was not an abnormal amount of fluid in the ventricles. The nervous substance was healthy, and the membranes in the upper part of head and cerebrum were apparently in a normal condition. All the evidence of disease was found in base of brain and cervical portion of the spinal cord. He was a healthy, athletic boy, very large for his age. Before refering to the therapeutic history of this disease we will briefly present some of the pathological deductions, and in so doing will consult the able monograph of Prof. JEWELL.

*First.* The symptoms which refer to the nervous system are always present; but in many cases they are much milder than in the two cases above.

*Second.* The pathological appearances found in the cerebro-spinal cavity are in *some degree* always present, and in all malignant cases in a marked degree.

*Third.* The monographs of Europe and this country, examined by Prof. JEWELL, teach that the blood shows a decided increase of fibrine, and generally of corpuscles. After death the blood is more fluid and dark than customary.

*Fourth.* It is not a form of typhus.

*Fifth.* It is not contagious, but probably infectious.

*Sixth.* It is caused by a special external epidemic poison.

*Seventh.* This supposed cause acts on the nervous system and blood, and destroys life by its septic or degenerative tendency in a few hours.

*Eighth.* The disease or its primary cause may be modified or influenced by malaria or periodical diseases.

With our present pathological knowledge, aided by the experience of able men in this country and Europe, what are the best indications for treatment?

The physician, when called to a malignant case, and sees the rapidity with which the nervous, vascular, and secretory function are destroyed, the blood looses its organic and vital power by the action of some morbid and energetic poison, which is, if not controlled, bound to quickly divorce soul and body. With our present knowledge of the disease we cannot fail to doubt the power of remedial agents to stop the mischief.

The indications for the treatment of this disease are:

*First.* To equalize the circulation, and thus prevent, if possible, local hyperemia, especially of the meningis of cerebellum and spinal cord.

*Second.* To quiet the irritability of the nervous system, and, if possible, introduce into the system some anteseptic which will neutralize or in some way counteract the deleterious effects of the epidemic poison, which is supposed to be the primary cause of the disease.

If the local hyperemia is not speedily removed the distension and extreme congestion of the capillaries of the membranes of the cerebellum and spinal cord will, as in the above case, result in effusion, and that within twelve hours. In these cases the plethora is asthenic, and experience does not teach us that blood-letting will recall the lost tone of the over-distended vessels. Our experience accords with a great majority of the observers of this disease, both in this country and Europe, that blood-letting is inadmissible. Local bleeding may in some cases do good, by cups and leeches along the spine.

The physician, when first called to a case, and finds a quick pulse; eyes suffused, and symptoms of great cerebral excitement, and apparently general congestion, is impressed with the belief that general blood-letting would be beneficial; but the weight of experience is against its employment. It seems to be pretty generally admitted that this remedy increases the sighing or gasping breathing, which warns us of the rapid failure of the spinal functions, which are so intimately connected with the processes of life.

The remedial agents which act as stimulants to the heart and vessels, and to the cerebral functions, and at the same time operate as sedatives to the medullary system, if energetically persevered in at the onset of the disease, offer some hope of equalizing the capillary circulation. These are alcohol, ether, opium, sumbul, chloroform, and many others, are acknowledged as useful and efficient in spasmodic and convulsive affections, when there is no inflammation.

They seem to give vigor and equal diffusion to the circulation, and thus prevent local determination and congestion of blood in the nervous centers. In this terrible disease it is advisable to assist these agents by the external application of heat and counter-irritation.

Dr. JEWELL says, after a thorough investigation of the treatment of this disease, "stimulants have been found useful in all periods of the disease, especially in proportion as they are early employed, both externally and internally."

Opium seems to stand at the head of remedial agents, and is emphatically and almost unanimously endorsed by those who have witnessed its effects in this disease.

If we succeed with our opium, brandy, and external applications in equalizing the circulation, and for the time, relieve the local congestion, if the primary or *epidemic cause* is not removed or neutralized, we may expect a return of the symptoms in all of their former vigor. This being true, it is certainly important that we promptly and early use those remedies which experience has pointed out as best calculated to fill the second indication for the treatment of the disease.

We have no knowledge of this non-cognizable or *zymotic* agent save in its effects, consequently the remedies best calculated to remove or neutralize it must be submitted to the test of experiment. It is supposed to be a leaven or ferment, an active septic matter, a very small quantity of which, when introduced into living organism, will promote change and decomposition. "A little leaven leaveneth the whole lump." Its action is probably chemical, and breaks up the affinities of compound organic principles. If this is true we have a reasonable explanation of the rapid decomposition and change this agent works in the blood. The remedies found most efficient in counteracting its effects when in the system, are the arsenites and permanganates; chlorine and charcoal are also highly recommended as capable of destroying this class (zomatic) poisons. Their beneficial effects are produced without as well as within the body. Chlorine may be used with great benefit on the floors and walls of infected houses; but its acrid qualities renders it objectionable as an internal remedy.

Would it not be well for future investigators to try and determine the true nature of this concealed epidemic cause, by frequent analyzation of the blood of those who contract the disease.

It is believed by many that epidemic and infectious diseases are caused by the invasion and operation of living parasites, which enter the body and disturb its functions and structures. If it is an animacule, may they, when introduced into the system, under certain circumstances, be capable of self-propagation. This idea receives support from the phenomena of itch. This disease infects through the itch-mite, and is known to spread by this animal's propagation.

It is a generally admitted fact that cerebro-spinal meningitis prevails in damp or foggy weather. Thus the subtle poison or animalcule seems to require moisture to aid it in its invisible march through space on its mission of death. This is all purely hypothetical, and with our present knowledge is not supported by any trustworthy facts.

It is our purpose in this paper to refer to all of the *most important* remedial agents which have been employed in the treatment of this fearful malady, and to give the weight of experience for and against their use. The *cause* being non-cognizable, we must appeal to the experience of careful and honest observers, in order to enable us to judiciously select our remedies.

Those who have had the most extended experience do not favor the free use of *mercurials.* There are but few who recommend *emetics.*

The use of *purgatives* are often indicated, but they should be of a mild, laxative character, and simply unload the bowels without producing a depressing effect.

The primary effect of *cold* applied to the head and spine has, in the hands of a large majority of observers, been attended with beneficial results. It should not be used until after reaction, and only when there is heat of the surface, and in decidedly febrile cases. In the first stage, or when there is chilliness this remedy is inadmissible.

We have not found *quinine* beneficial, unless there is a decided periodicity in the disease. Prof. JEWELL has examined many authorities, and gives it as his opinion that a large majority are against its use. Drs. NOBLE, WALES, and PALMER, are of the opinion that *strychnine*, when early employed, is a valuable remedial agent.

In the preparation of this paper we have not aimed at giving more than a general outline of the nature and treatment of this fearful malady. It would extend it far beyond the patience of the members of this society, if we were to enter into the details of treatment. Therefore, these remarks are respectfully submitted to your consideration.

